# A Fast Transient Absorption Study of Co(AcAc)_3_

**DOI:** 10.3389/fchem.2019.00348

**Published:** 2019-05-21

**Authors:** Luisa Ferrari, Mauro Satta, Amedeo Palma, Lorenzo Di Mario, Daniele Catone, Patrick O'Keeffe, Nicola Zema, Tommaso Prosperi, Stefano Turchini

**Affiliations:** ^1^CNR-ISM, Division of Ultrafast Processes in Materials (FLASHit), Area della Ricerca di Roma Tor Vergata, Rome, Italy; ^2^CNR-ISMN, Chemistry Department, Università di Roma Sapienza, Rome, Italy; ^3^CNR-ISMN, Area della Ricerca di Roma 1 - Montelibretti, Rome, Italy; ^4^CNR-ISM, Division of Ultrafast Processes in Materials (FLASHit), Area della Ricerca di Roma 1 - Montelibretti, Rome, Italy

**Keywords:** fast transient absorption, TDDFT (time-dependent density functional theory) calculations, femtosecand laser pulses, metal complexes, charge - transfer

## Abstract

The study of transition metal coordination complexes has played a key role in establishing quantum chemistry concepts such as that of ligand field theory. Furthermore, the study of the dynamics of their excited states is of primary importance in determining the de-excitation path of electrons to tailor the electronic properties required for important technological applications. This work focuses on femtosecond transient absorption spectroscopy of Cobalt tris(acetylacetonate) (Co(AcAc)_3_) in solution. The fast transient absorption spectroscopy has been employed to study the excited state dynamics after optical excitation. Density functional theory coupled with the polarizable continuum model has been used to characterize the geometries and the electronic states of the solvated ion. The excited states have been calculated using the time dependent density functional theory formalism. The time resolved dynamics of the ligand to metal charge transfer excitation revealed a biphasic behavior with an ultrafast rise time of 0.07 ± 0.04 ps and a decay time of 1.5 ± 0.3 ps, while the ligand field excitations dynamics is characterized by a rise time of 0.07 ± 0.04 ps and a decay time of 1.8 ± 0.3 ps. Time dependent density functional theory calculations of the spin-orbit coupling suggest that the ultrafast rise time can be related to the intersystem crossing from the originally photoexcited state. The picosecond decay is faster than that of similar cobalt coordination complexes and is mainly assigned to internal conversion within the triplet state manifold. The lack of detectable long living states (>5 ps) suggests that non-radiative decay plays an important role in the dynamics of these molecules.

## Introduction

Transition metal complexes are of paramount importance in chemical and biological photochemical processes (Ruggiero et al., 2014) involving the conversion of the energy of visible light into chemical energy and the activation of redox-states for catalysis (Prier et al., 2013). Indeed, it is desirable to study the early formation of optically excited states, to explore the possibility of triggering and tailoring the electronic and structural properties of this class of compounds. Moreover, the spreading of the population of the excited electrons into different relaxation channels plays a key role in smart sensors and ultrafast devices (Wada, 2004).

The development of femtosecond laser sources gave rise to a revolution in the comprehension of the fate of excited electrons and provided a microscopic basis for already established theories such as molecular internal vibration relaxation (Grossmann, 2013), mostly based on the analysis of the photo fragmentation processes (Wada and Tanaka, 2013). Moreover, the advent of ultrafast X-ray facilities provided new insight into electronic and structural dynamics of transition metal complexes (Huse et al., 2011; Chergui, 2018). To this end future perspectives are strongly related to the ultrafast application of VUV-Soft X-ray PhotoElectron Spectroscopy studies in the gas phase, to our knowledge still lacking in the field of metal complexes, and the application of theoretical state of the art methods to characterize the dynamics of the electronic structure (Squibb et al., 2018).

The dynamics studies usually are modeled by excitation following the Franck-Condon approximation, which takes place in about 1 fs (Demtröder, 2008), then three different relaxation processes occur (Tramer et al., 2010). Internal conversion (IC) allows the de-excitation to a different electronic state within the same spin multiplicity. Intersystem crossing (ISC) is the mechanism characterized by a change of spin of the relevant electronic states. IC and ISC are first-order perturbation theory processes, their decay mode can be modeled by exponential functions and their rate is inversely proportional to the energy difference of the involved states (Bixon and Jortner, 1968). Vibrational cooling (VC), which is usually the fastest process, represents the de-excitation from the highest excited to the lower vibrational levels within the same electronic state; the decay is generally associated with energy dissipation toward the surrounding medium and can be modeled by perturbation theory using a bath Hamiltonian (Fujisaki et al., 2006).

Transition metal complexes present a manifold of low-lying excited states with a high density of states that enables them to efficiently harvest light; in a narrow interval of energy (hundreds of meV) the excited electronic states display different electronic character, inducing a great variety of de-excitation pathways and reactivity processes after optical excitation. Moreover, in transition metal compounds the spin-orbit coupling term of the Hamiltonian is strong because of the open d-shell of the metal and it favors a fast ISC with the subsequent formation of long living states. This is in contrast to organic molecules where ISC is generally slower than IC (Tramer et al., 2010). The strong spin-orbit term alters the hierarchy of the perturbations and makes the distinction between ISC and IC fuzzier. It is reasonable to assume that these two processes could occur in the same time scale and, consequently, convoluted, although in a quantum-mechanical picture the relative strength between the non-Born-Oppenheimer terms and the spin-orbit does not allow us to consider the two processes separated in terms of perturbation theory. The presence of ISC is generally associated with long living states that de-excite by phosphorescence decay.

The existence of two time scales related to fast and slow relaxation processes reflects different photochemical and photophysical phenomena. The fast evolution after photoexcitation displays intramolecular processes that dramatically change the electronic and structural properties in a very selective pathway; this can be tailored by tuning the excitation energy and the vibrational composition of the excited state. On the other hand, relaxed fully thermalized excited states with lifetimes greater than the diffusion time scale provide the basis for slow photochemistry reactions that are not thermodynamically accessible in the ground state. Moreover, the electronic character of the excited states provides different chemical selectivity with respect to the ground state.

In metal complexes the tailoring of ISC by tuning the excitation channel can dramatically change the ratio between the fluorescence and phosphorescence relaxation channels (Hsu et al., 2012), with potential applications in sensor design.

The relevance of ISC in transition metal complexes dynamics has been extensively studied (Vlček, 2000; McCusker, 2003; Wagenknecht and Ford, 2011). Fluorescence up-conversion measurements on [Ru(bpy)_3_]^2+^ display ISC for metal to ligand charge transfer (MLCT) states: ^1^MLCT → ^3^MLCT with τ = 40 ± 15 fs, while IC displays a time scale of the order of picoseconds (Bhasikuttan et al., 2002). On the other hand, fast transient absorption spectroscopy (FTAS) measurements assign a τ≈100 fs for the overall formation of the ^3^MLCT (Damrauer et al., 1997). [Fe(II)(tren(py)_3_)]^2+^ shows ISC characteristic time constant less than 1 ps (Monat and McCusker, 2000). To establish the ^5^T_2_ character of the long living state, the difference of absorption spectra of Fe(II) complexes with ^5^T_2_ (high-spin) and ^1^A_1_ (low spin) ground state provided a reference for the time-resolved excited state spectrum. The assignment of ^5^T_2_ to ^1^A_1_ conversion was recently found by a variable temperature FTAS study of [Fe(bpy)_3_]^2+^-type complexes and [Fe(terpy)_2_]^2+^ (Carey et al., 2019). FTAS studies on Co(III) compounds have been presented (McCusker et al., 1993): [Co(en)_3_](ClO_4_)_3_, [Co(tpen)](C1O_4_)_3_, and [Co(tppn)](C1O_4_)_3_ show biphasic relaxation kinetics; in the case of [Co(tpen)]^3+^ excited-state decay is expressed by two time constants τ_1_ = 4 ± 2 ps and τ_2_ = 44 ± 5 ps, for [Co(tppn)]^3+^ τ_τ_ = 3 ± 1 ps and τ_2_ = 51 ± 3 ps, while the data for [Co(en)_3_]^3+^ give τ_τ_ = 2 ± 1 ps and τ_2_ = 450 ± 100 ps. For [Co(tpen)](C1O_4_)_3_ and [Co(tppn)](C1O_4_)_3_ the experimental data suggest a lifetime of the ^1^LMCT (ligand to metal CT) state less than 1 ps. No fluorescence yield was observed for the three compounds. Regarding the kinetics of the non-radiative decay, the relation between photochemical properties and ligand field excited state character leads to the assignment of the lowest lying excited state as ^5^T_2_ for the octahedral Co(III) complexes considered. Cr(III)(AcAc)_3_ has been thoroughly studied by means of FTAS (Juban and McCusker, 2005) and shows the formation of the ^2^E_2_ state from LMCT and ligand-field excitation. The ligand field excitation presents a monophasic decay of τ_1_ = 1.1 ± 0.1 ps for every excitation and pump wavelength, LMCT excitation exhibits τ_τ_ = 50 ± 20 fs and τ_2_ = 1.2 ± 0.2 ps, associated with charge transfer to ligand field manifold ISC and VC to ^2^E_2_, respectively. Transient infrared spectroscopy points out that 70-85% of the ground state population of Cr(acac)_3_ recovers with a time constant of 15 ps, and the remaining population is described by the lifetime of the ^2^E state (Maçôas et al., 2007).

These results clearly indicate the action of fast intersystem crossing and reverse the usual sketch of ISC being the slowest process.

We present a FTAS study of Co(AcAc)_3_, where AcAc is the Acetylacetone molecule, a diketone moiety which forms complexes with transition metal atoms. This is a transition metal complex currently employed as a catalyst for the formation of C–C bonds and oxidation reactions (Ishii et al., 1996). This compound has closed shell with *D*_3_ symmetry point group and allows reliable and accurate quantum chemistry calculations. The electronic structure of the molecule was characterized by photoelectron and circular dichroism photoelectron spectroscopy (Catone et al., 2012, 2013) with good agreement of theory with spectroscopic data.

By means of density functional theory (DFT) and time dependent density functional theory (TDDFT) quantum chemistry calculations, we provide a picture of the structural and electronic properties involved in the ultra-fast dynamics, discussing the ligand-field and the charge transfer states excitation. Ligand-field approach is very important for a qualitative assignment of electronic character and symmetry of excited states. DFT and TDDFT calculations provide a more precise characterization of the ligand to metal and metal to ligand excited states. FTAS data will be discussed in view of these results.

The aim of the present work is to investigate the interplay between ISC and the other relaxation processes on the basis of the calculated electronic structure and spectroscopic data.

## Experimental Details

FTAS is a pump-probe methodology that measures the difference in absorption between the excited state and the ground state taken at different time delays after the optical excitation at a defined wavelength.

The pump was generated by an optical parametric amplifier (OPA) fed by the 800 nm radiation of an amplified Ti:Sapphire laser with a pulse length of 35 fs and a repetition rate of 1 kHz, and the probe was a white light supercontinuum generated in a commercial transient absorption spectrometer (FemtoFrame II-IB photonics). The probe wavelengths ranged between 400 and 750 nm while the pump-probe delay time was scanned up to 100 ps, with an overall temporal instrument response function described by a Gaussian peak with FWHM of 50 fs. Co(AcAc)_3_ (Sigma Aldrich, purity 99.99%) was diluted in acetonitrile (ACN). Two different concentrations were used in the pump-probe experiments on Co(AcAc)_3_: 0.01 M (excitation wavelength 365 and 390 nm) and 0.05 M (excitation wavelength 580 and 650 nm). Typical pump laser fluence was 500–2500 μJ/cm^2^. The measurements were carried out in a quartz cell with path length 1 mm. A blank pump-probe experiment was performed to seek non-linear contributions of the solvent in the experiment. We paid attention to minimize cross-phase modulation structures in the transient spectrum by choosing a suitable pump fluence, although for low pump absorption it was not possible to eliminate it completely. The chirp of the FTAS signal was corrected by an alignment based on a polynomial fit. Since the transient absorption slowly varies as a function of the wavelength, to increase the signal to noise ratio the temporal cuts were averaged ±5 nm around the chosen wavelength. No FTAS signal was detected in the 800–1600 nm region of probe wavelength. Measurements were taken at the magic angle between the linear polarization vectors of pump and probe radiation. Further details of the experimental set-up can be found in previous publications (Catone et al., 2018; Fratoddi et al., 2018).

## Computational Details

The description of the geometries and of the electronic structures of the gas phase and of solvated complex has been carried out using DFT formalism coupled with the polarizable continuum model (PCM) based on reaction field calculations and the integral equation formalism (Tomasi et al., 2005; Scalmani and Frisch, 2010). The excited states have been studied within the TDDFT approach (Bauernschmitt and Ahlrichs, 1996). The hybrid exchange-correlation functional Becke (Becke, 1993), three-parameter, Lee-Yang-Parr (Vosko et al., 1980; Lee et al., 1988; Stephens et al., 1994) has been adopted together with the split-valence double-zeta Pople basis set (Hariharan and Pople, 1973; Francl et al., 1982) with the addition of extra functions as established by Barone et al. (Barone et al., 2008) to be used in the frame of effective discrete/continuum solvent models. TDDFT and the chosen basis set are a good compromise between accuracy and computer time consuming for these complexes. TDDFT with B3LYP has been successfully used in the literature to characterize the electronic structure in similar calculations (Savarese et al., 2012, 2014; Catone et al., 2018).

The geometries were fully optimized for the ground states of the singlet, triplet and quintet spin configurations; at each of these geometries the first twenty excited electronic states have been computed. In order to have a picture of the excitation of the singlet, triplet and quintet states during the vertical transition from the initial geometry of the ground state, we have calculated the singlet, triplet, quintet excited electronic levels at the singlet minimum energy geometry. All the calculations were performed using the Gaussian code (Frisch et al., 2016).

The ISC processes have been described by estimating the relative non-radiative lifetimes computed by means of spin-orbit coupling matrix elements. In particular, we have used ADF (2018) code to calculate the spin-orbit coupling as a perturbation to a scalar relativistic calculation of TDDFT excitation energies for the gas phase. The TDDFT has been performed using the B3LYP functional, in analogy with our results obtained by Gaussian code, with double zeta polarization basis set on H, C, and O atoms, whereas the Co basis set is double zeta with 1s, 2s, 2p frozen core.

The number of singlet excited states considered was 20 for the singlet, and 60 for the triplet due to degeneracy. The spin-orbit coupling matrix was computed at the geometry corresponding to vertical transition from the equilibrium geometry of the electronic ground state. Several unsuccessful attempts to follow the geometry relaxation of the singlet electronic excited states were performed resulting in several singlet-singlet crossings along the optimization paths which at the end always lead to the first singlet excited state. The spin-orbit coupling matrix was computed at five different geometries only for the first singlet excited state, taking the geometries from the optimization of the first singlet excited state performed by Gaussian code.

The ISC non-radiative lifetimes $\tau_{i}^{NR}$, where *i* is the ith singlet excited state, have been computed following the Fermi golden rule with a Franck-Condon weighted density of states (FCWD), which assumes an equal vibrational structure between the involved electronic states (Valiev et al., 2018):

$$1/\tau_{i}^{NR}=\frac{2\pi }{\hbar }\sum_{j}SO_{ij}^{2}\frac{\Gamma }{\left(\Delta E_{ij}^{2}+\frac{\Gamma^{2}}{4}\right)}$$

with the relaxation width of the vibronic levels Γ taken as 10^14^ s^−1^, which is a condition generally fulfilled in experimental studies of luminescence properties (Valiev et al., 2018), SO_ij_ are the spin- orbit matrix element coupling the ith singlet state with the jth triplet state, and ΔE_ij_ is the corresponding energy difference.

## Results and Discussion

**Figure 1:**
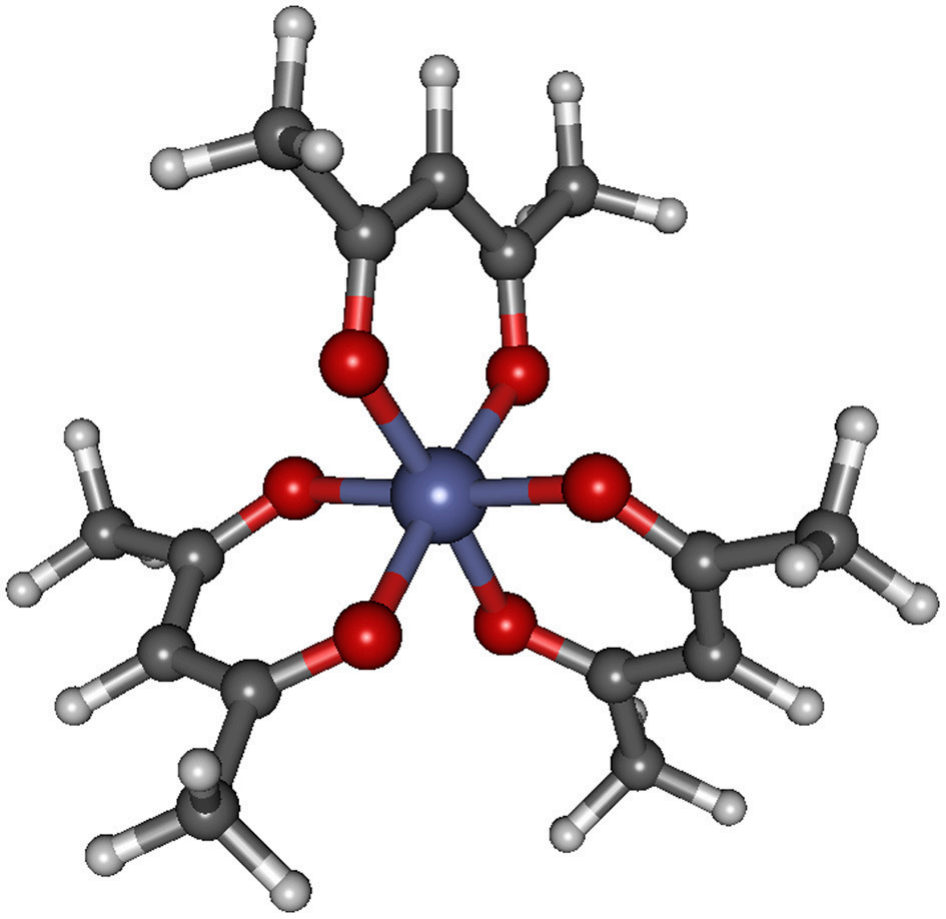
Geometrical structure of Co(AcAc)_3_. Bond lengths and angles are reported in Table 1 for the singlet, triplet and quintet ground state minimum energy for both gas phase and ACN solvent.

The structure of the Co(AcAc)_3_ complex is shown in Figure 1, while the most significant geometrical parameters are reported in Table 1 for the minimum energy geometries of the three spin configurations.

**Table 1:** Most significant geometrical parameters for the singlet, triplet and quintet ground state minimum energy and first excited state singlet level for Co(AcAc)_3_ in the gas phase and in ACN solvent.

	**Gas phase**				**Acetonitrile**			
	**Ground state singlet**	**Ground state triplet**	**Ground state quintet**	**First excited state singlet**	**Ground state singlet**	**Ground state triplet**	**Ground state quintet**	**First excited state singlet**
Co-O	1.91	2.03–1.91	2.01	1.91–2.08	1.91	1.90–2.07	2.01	1.91–2.07
O-Co-O_(ring)_	95.2	88.4–93.4	86.3–86.4	89.8–93.9	95.2	88.1–93.6	86.4	88.1–93.6
C-O	1.27	1.26–1.27	1.27	1.26–1.27	1.27	1.27–1.28	1.27	1.27–1.28
C_sp2_-C_sp2_	1.40	1.41	1.40	1.41	1.40	1.41	1.40	1.40–1.41
C_sp2_-C_sp3_	1.51	1.51	1.51	1.51	1.51	1.51	1.51	1.51
C-O-Co-O	0.0	4.5–25.1	0.5–1.0	1.0–21.8	0.0	7.5-25.0	0.5–1.8	7.1–26.5

*Distances in Angstrom, angles in degrees*.

**Figure 2:**
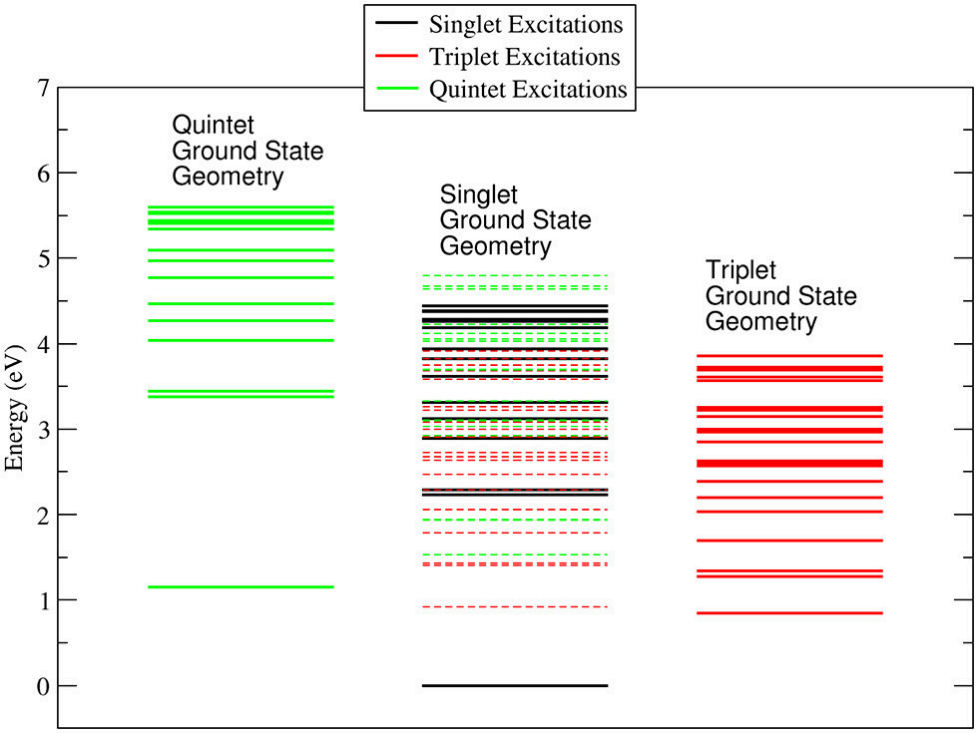
Energy levels calculated for the singlet, triplet and quintet spin configuration of Co(AcAc)_3_ in the gas phase. In continuous lines are reported the energy levels calculated at the minimum energy geometry of each ground state. In dashed lines are indicated the triplet and quintet excited energy levels evaluated at the geometry of the singlet ground state minimum energy.

It is worth noting that while in the singlet and quintet configuration the three rings are all planar and the symmetry of the system remains D_3_, in the triplet case one of the rings is bent ( C-O-Co-O 25.1° and 25.0° for the gas phase and ACN solvent, respectively) lowering the symmetry of the system. The minor changes in the geometrical parameters reported in Table 1 for the three different spin configurations should take place during the ISC relaxation dynamics following the initial excitation. In the case of the gas-phase complex the calculations show (see Figure 2) that the triplet ground state is about 1 eV above the singlet ground state, while the quintet ground state is slightly higher than the triplet state. For the triplet spin configuration (right part of Figure 2) the energy level distribution is quite uniform: about 20 levels in an energy interval of 3 eV. In contrast, for the quintet spin case (left part of Figure 2), there is an energy gap of about 2 eV between the ground and first excited state, above which there is a quite uniform distribution of the density of states (DOS) up to 5.5 eV. In order to have a qualitative picture of the dynamics involving ISC, we report the singlet, triplet and quintet excited energy levels calculated at the geometry of the singlet ground state minimum energy (central part of Figure 2). In the energy gap between the first singlet excited state and the ground state energy (about 2 eV), there are several other energy levels of the triplet and quintet states. An analogous quite high DOS is calculated up to 5 eV above the ground singlet state, thus supporting the possibility of a complex intersystem evolution of the wavepacket produced by the initial vertical photo-excitation.

**Figure 3:**
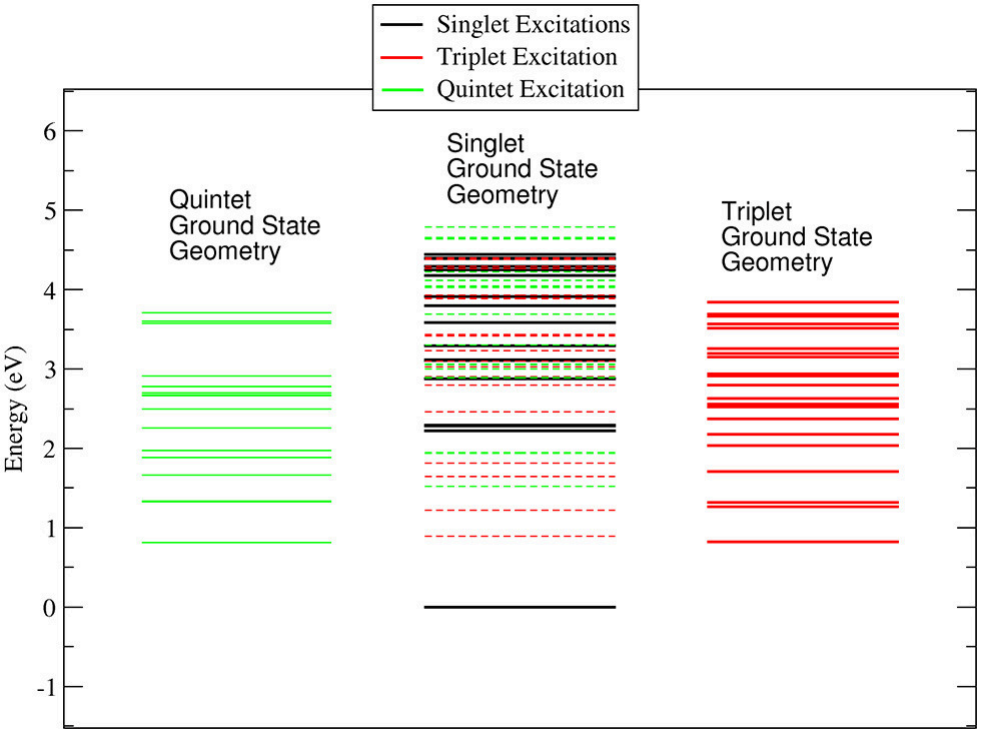
Energy levels calculated for the singlet, triplet and quintet spin configuration of Co(AcAc)_3_ in ACN solvent. In continuous lines are reported the energy levels calculated at the minimum energy geometry of each ground state. In dashed lines are indicated the triplet and quintet excited energy levels evaluated at the geometry of the singlet ground state minimum energy.

In the ACN solvent (see Figure 3) the quintet ground state minimum energy is lower than that of the triplet, and it is about 0.8 eV above the singlet ground state. The DOS for the singlet, triplet and quintet states are similar to each other, in particular the DOS calculated in the geometry of the singlet ground state minimum energy are quite similar to those calculated in the gas phase, while for quintet the density of state changes significantly. The large difference between the quintet energy calculated in the gas phase and ACN is related to the high polarizability of these spin states with respect to triplets and singlets.

**Figure 4:**
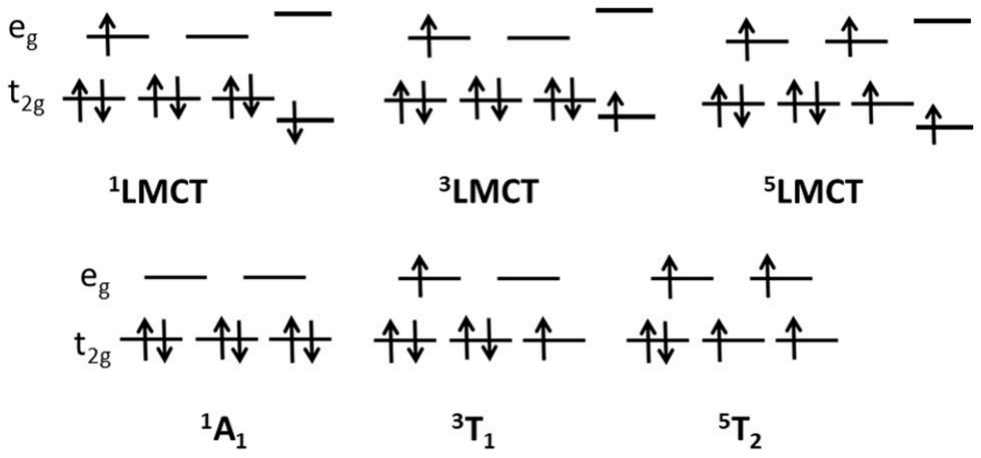
Schematic diagram of one electron excited states for a d^6^ orbital configuration in an octahedral field (lower row) and LMCT states with the transfer of one electron from the ligand to the metal states (upper row).

The electronic scheme of octahedral metal complexes is ruled by ligand field splitting of the d electrons. Tanabe-Sugano d^6^ diagrams assign ^1^A_1_ character to the ground state, with excited states in order of energy ^3^T_1_, ^3^T_2_, ^1^T_1_ (Griffith, 1961). The energy of the ^5^T_2_ with respect to singlet and triplets states depends on the ratio Δ/B of the crystal field parameters, where Δ is the octahedral energy splitting and B the Racah electron repulsion parameter. Figure 4 reports the one electron picture of the ^1^A_1_, ^3^T_1_, ^5^T_2_ octahedral ligand field states along with the excited states involving the ligand. The D_3_ point group symmetry splits the above octahedral states, however in this work we shall relate each state to the original octahedral symmetry in order to simplify comparison with the literature.

**Figure 5:**
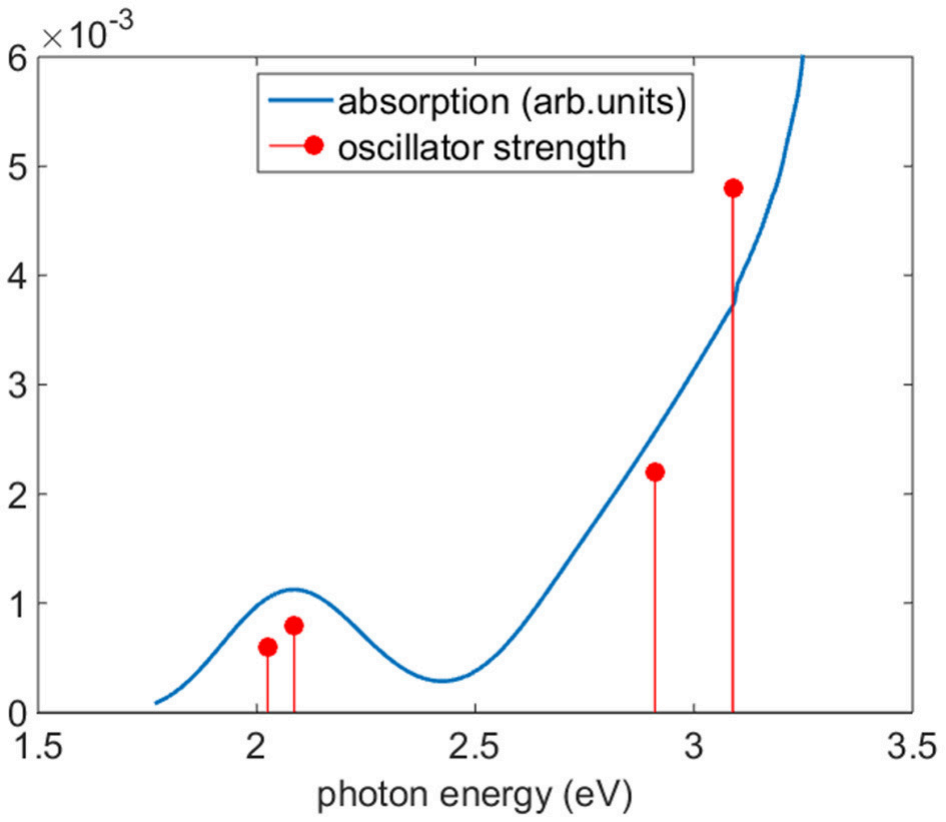
Experimental absorption (blue line) of Co(AcAc)_3_ and oscillator strengths (red circles). The calculated excitation energies have been shifted of −0.2 eV to improve the accord with experimental data. The oscillator strengths of quasi-degenerate states have been summed.

Figure 5 reports the experimental absorption of Co(AcAc)_3_ along with the theoretical oscillator strength calculations in ACN. The spectrum shows a broad band centered at 2.1 eV, where the d-orbital excited states of the Co are relevant, and a steep rise starting at 2.4 eV, associated with the LMCT manifold. Table 2 displays the TDDFT excitation energies, the irreducible representations and oscillator strengths calculated for ACN; the numeric label in the table will be used in the discussion to identify the states. In order to have a good agreement with the experiment a rigid shift of 0.2 eV toward lower energies is applied to the calculated data. This shift value is compatible with the accuracy of the TDDFT method. The oscillator strengths of quasi-degenerate states have been summed.

**Table 2:** Theoretical transition energies, wavelengths and oscillator strengths of optically excited states for Co(AcAc)_3_ in ACN.

**Excited state (Singlet)**	**Symmetry**	**Energy**		**Oscillator strength**
		**(eV)**	**(nm)**	
1	1 ^1^A	2.2250	557.23	0.0006
2	1 ^1^E	2.2854	542.51	0.0004
3	1 ^1^E	2.2876	541.98	0.0004
4	2 ^1^A	2.8762	431.07	0.0000
5	2 ^1^E	3.1103	398.62	0.0011
6	2 ^1^E	3.1118	398.43	0.0011
7	3 ^1^E	3.2884	377.04	0.0024
8	3 ^1^E	3.2916	376.66	0.0024
9	3 ^1^A	3.5876	345.59	0.0000
10	4 ^1^E	3.7985	326.40	0.0001

In the photon energy range 1.8-2.7 eV the envelope of d-d transitions is reproduced by the transition to excited states 1-3. The octahedral ^1^T_1_ state is split by the D_3_ symmetry group into an A state (1) and double degenerate E states (2,3) at calculated excitation energies 2.23 and 2.29 eV, respectively. The charge transfer states ^1^LMCT are represented by an A state at 2.88 eV (4), associated with zero oscillator strength, and a pair of doubly degenerate E states at 3.11 eV (5,6) and 3.28 eV (7,8), respectively.

**Figure 6:**
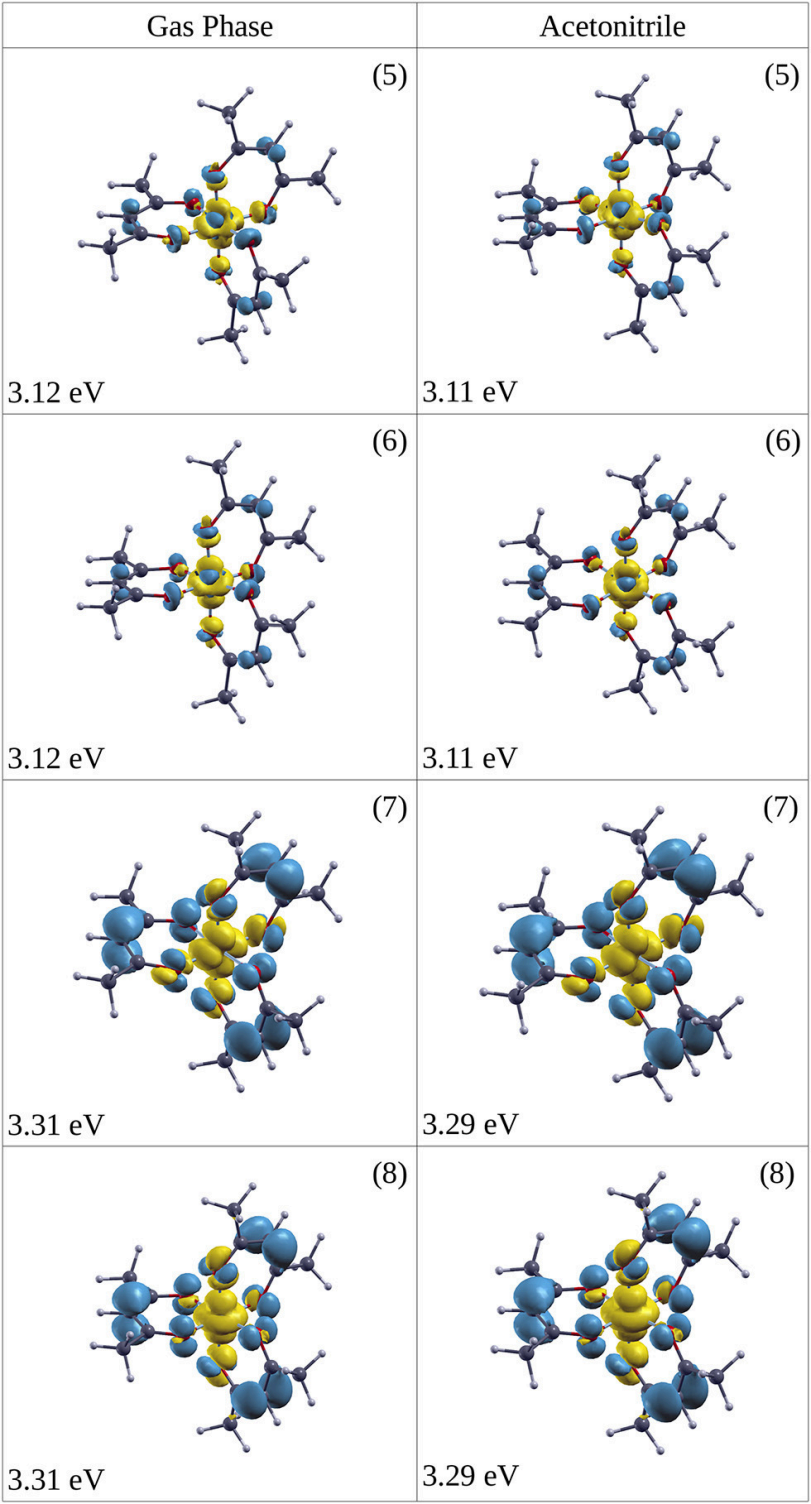
Iso-surface of the change in electron densities upon vertical transitions of optically excited states (singlet) for Co(AcAc)_3_ in the gas phase (left side) and ACN solvent (right side). In yellow the positive value iso-surface are represented, while with the blue are indicated the negative value ones. The iso-surface threshold is fixed to 0.0025 a.u. The energy of the excitation and the numeric label of the state (see Table 2) are reported.

The excitation (^1^A_1_→^1^LMCT) produces a marked ligand-to-metal character for the complex both in the gas phase and solvated in ACN, as Figure 6 shows (excitation at 5 (3.11 eV), 6 (3.11 eV), 7 (3.29 eV), 8 (3.29 eV) in ACN), where there is a charge transfer from the three organic ring molecules of AcAc to the cobalt central atom. This electronic rearrangement does not invert the net charge transfer which occurs in the ground state of the metal complex, where about 0.5 *e* of charge is transferred from the metal atom to the ligands. The yellow iso-density surfaces represent an enrichment of the electron charge density upon excitation, whereas the blue iso-surfaces indicate a depletion in the electronic charge density, as reported in Figure 6: in particular there is a rearrangement of the electron charge density over the p MOs of the oxygen atoms, while the CH groups are net charge donors during this electronic transition with a reduction in the p MOs charge density. The excitation at 3.11 eV (399 nm) produces a charge electron density shift from the rings to a $d_{z^{2}}$ MO of the central Co atom both in the gas phase and in the ACN solvent (see Figure 6). In one case of excitation at 3.29 eV (excitation 7 in Table 2) the charge density is shifted into a d_xy_ MO of Co, while in the other excitation at 3.29 eV (excitation 8 in Table 2) the density shift is from the ring to a $d_{z^{2}}$ MO of Co. The amount of the charge density shift is higher in the transition at 3.29 eV (377 nm) with respect to that at 3.11 eV (399 nm). Since it is difficult to exactly locate the energy position of the ^1^LMCT in the experimental spectrum, we chose two wavelengths for the pump to excite electronic states with reasonable excess energy: 5 and 6 at 390 nm (photon energy 3.18 eV) and 7 and 8 at 365 nm (photon energy 3.40 eV). For the excitation of the ligand-field state two wavelengths were chosen for the pump: 580 nm (2.13 eV) near the maximum of the ligand-field state absorption and 650 nm (1.91 eV) at the onset of the absorption curve.

**Figure 7:**
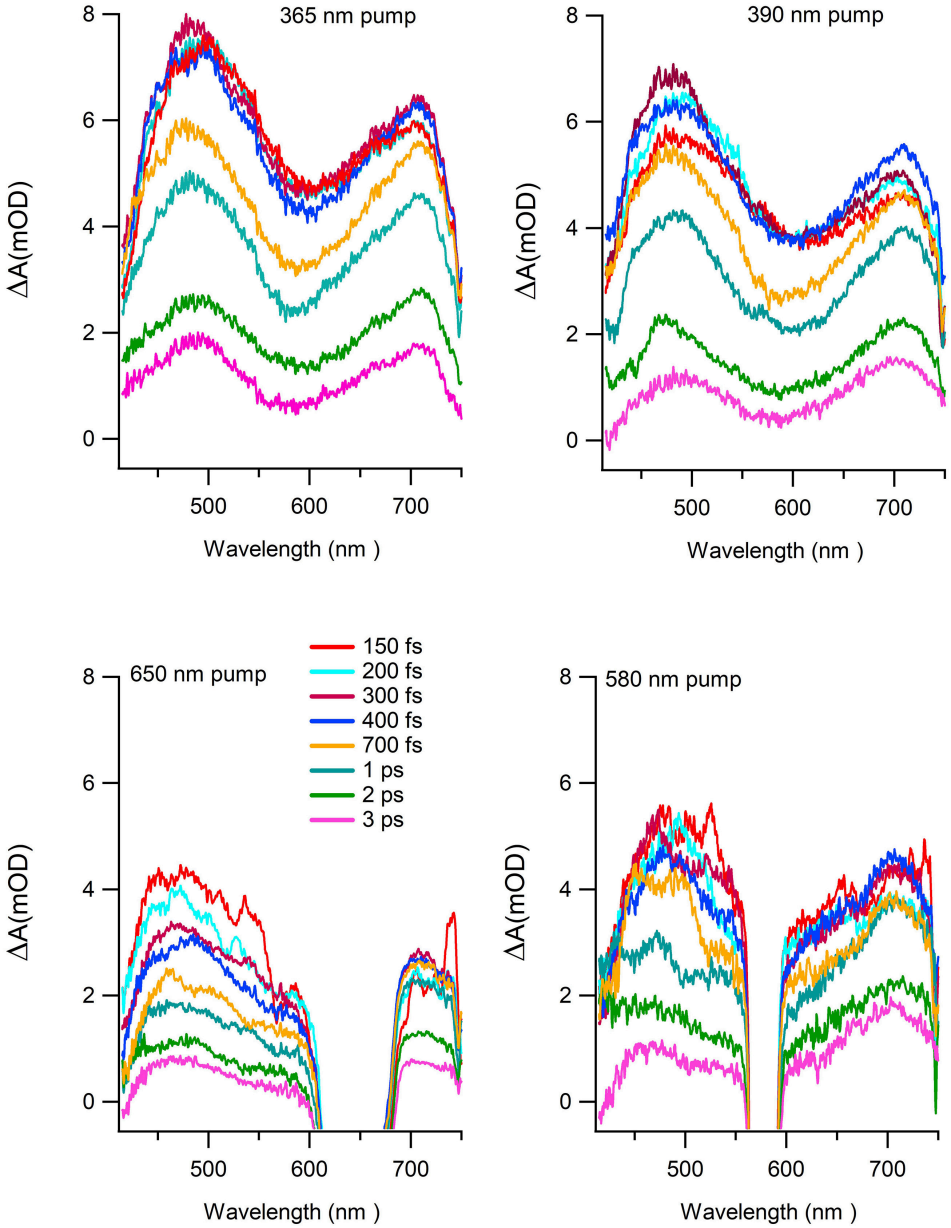
TA spectra as a function of wavelength at different delay times for the following excitation wavelength: 365 nm (upper left panel), 390 nm (upper right panel), 650 nm (lower left panel), 580 nm (lower right panel).

**Figure 8:**
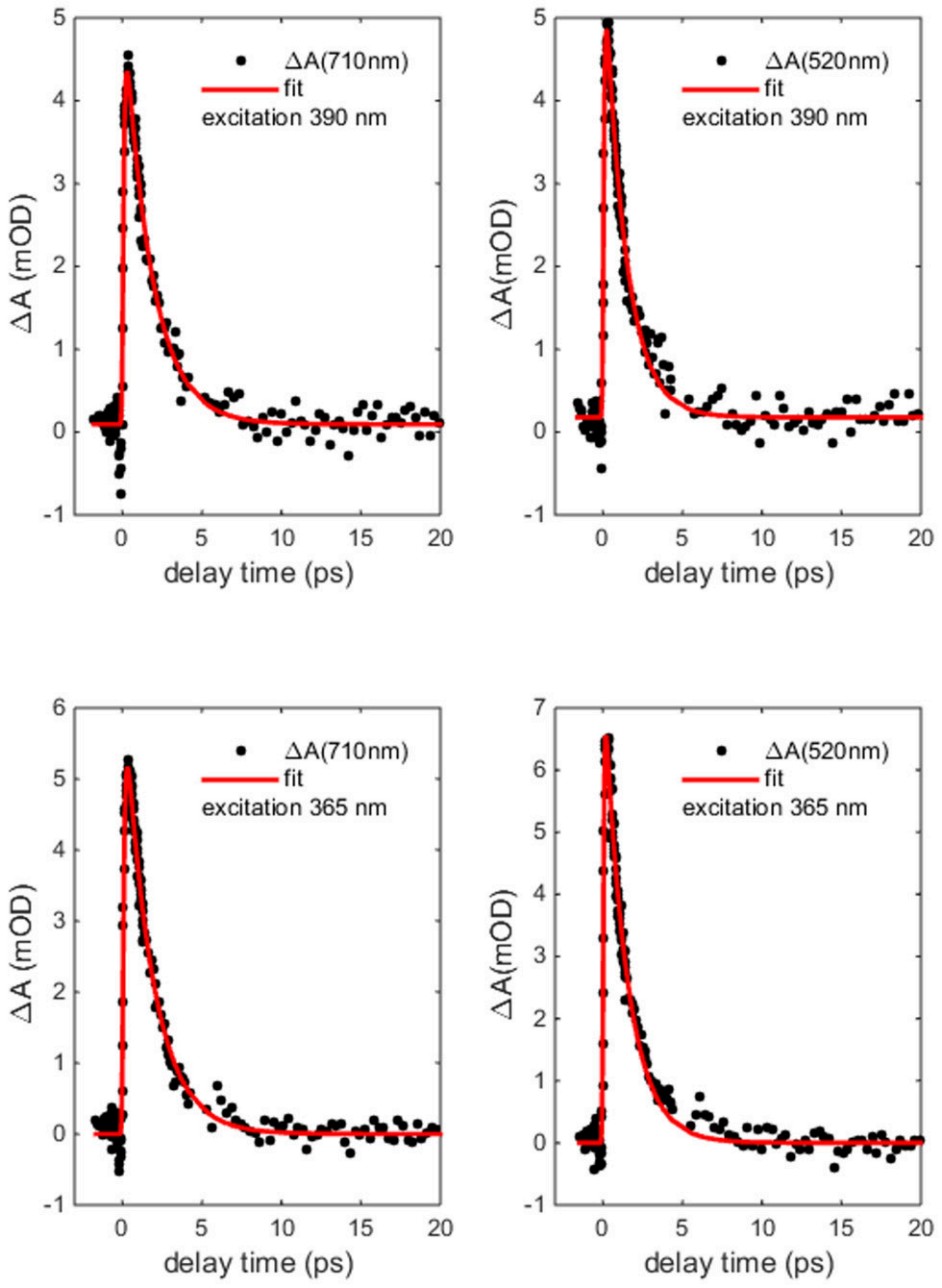
Co(AcAc)_3_ transient absorption (ΔA) dynamics at probe wavelength 520 and 710 nm as a function of delay time after 365 and 390 nm excitation (points) together with the biphasic exponential fit (solid line).

Figure 7 reports the transient absorption (TA) spectra (ΔA in the figure) in the 400-750 nm probe wavelength range at different delay times after the excitation at the chosen wavelength. The spectra taken at 365 and 390 nm (Figure 7 upper panels) display similar shape and time dependent behaviors. The TA spectra show positive values associated with a dominant contribution of the excited state absorption and two clearly defined broad bands, approximately centered at 480 and 700 nm. Two distinct time dependent regimes can be identified by analyzing the temporal evolution. Although the bands do not evolve in shape and wavelength position as a function of time, there is a clear evolution in their branching ratio. In the time range 0.1–1 ps the branching ratio between the maxima of the two structures monotonically decreases from roughly 1.3 to 1 and remains almost constant for *t*>1 ps. The change in absorption suggests that the formation time of the population of the electronic state, which decays to the ground state, is about 1 ps. The lack of the appearance of new structures and of meaningful shifts of the maxima, together with the recovery of the kinetic traces back to ΔA = 0, are strong indications that the molecule preserves its electronic structure and no photodegradation occurs. The dynamics of the Co(AcAc)_3_ fades out at about 5 ps and long living state are absent at every pump wavelength employed. Figure 8 reports the time evolution of the TA spectra at fixed probe wavelengths (520 and 710 nm) as a function of the delay time for excitations at 365 and 390 nm. The cuts are characterized by a swift rise, clearly revealed within the temporal resolution of the pump-probe system.

The curves were fitted with a biphasic exponential function one related to the ultrafast rise time and the other related to the slower picosecond relaxation process. In the fit the temporal instrument response function (50 fs) was taken into account. In Table 3 the time constants of the two processes are reported as a function of selected probe energies around the maxima of the two broad structures. For both pumps at 365 and 390 nm the time constants only very weakly depend on the probe wavelengths and substantially agree within the fit error. For the 390 nm excitation the averaged fit values are τ_1_ = 0.08 ± 0.04 ps for the rise and τ_2_ = 1.5 ± 0.3 ps for the decay, and for 365 nm excitation the value are τ_1_ = 0.07 ± 0.04 ps and τ_2_ = 1.5 ± 0.3 ps.

**Table 3:** Lifetimes after 365, 390, 580 and 650 nm excitation at selected probe wavelengths.

**Pump wavelength (nm)**	**Probe wavelength (nm)**	**τ_1_**	**τ_2_**
		**(ps)**	**(ps)**
365	460	0.08 ± 0.04	1.5 ± 0.2
	520	0.04 ± 0.04	1.3 ± 0.2
	680	0.08 ± 0.04	1.6 ± 0.2
	710	0.10 ± 0.04	1.7 ± 0.2
390	460	0.10 ± 0.04	1.5 ± 0.2
	520	0.06 ± 0.04	1.2 ± 0.2
	680	0.07 ± 0.04	1.7 ± 0.2
	710	0.10 ± 0.04	1.7 ± 0.2
580	460	-	1.6 ± 0.2
	520	-	1.6 ± 0.2
	680	0.06 ± 0.04	2.0 ± 0.2
	710	0.07 ± 0.04	2.1 ± 0.2
650	460	-	1.7 ± 0.2
	520	-	1.3 ± 0.2
	680	-	-
	710	0.07 ± 0.04	2.1 ± 0.2

*τ_1_ and τ_2_ are related to the lifetime of the rise and of the decay of the photoinduced absorption at the indicated wavelength, respectively. The rise is within the temporal resolution after 580 and 650 nm excitation for the 460 and 520 nm probe wavelength. After 650 nm excitation at the probe wavelength of 680 nm the fit of the lifetime was not possible due to the residual intensity of the pump*.

We now connect the phenomenological data analysis to a model of the dynamics of the Co(AcAc)_3_ LMCT excited states and assign the hierarchy of the processes governing the dynamics of the molecule excitation. According to the literature ISC is the most likely fastest process. A previous FTAS study of Co(III) compounds (McCusker et al., 1993) proposed two mechanisms to understand the possibility to access different spin configurations. The first mechanism is ISC, while the second one points out that the LMCT transition alters the charge of the Co(III) ion that assumes a Co(II) character. Due to known instability of Co(II) low-spin states the metal ion could provide a swift transition low-spin → high-spin. Both processes lead to the same final state: in the former case the mechanism has a molecular nature, in the latter is based on the metal ion.

In view of these results we suggest that the origin of the rise time lies in an ISC that takes place on time scale of the order of tens of fs (Bhasikuttan et al., 2002) which depopulates the ^1^LMCT in favor of different spin states. The sum of the manifold of these processes gives rise to the phenomenological fast onset. It is worth noticing that the variety of the states involved causes different probed dynamics that is reflected in the slightly different response of the states to the photon energy of the probe pulse. Hence the spread of the τ_1_ values is related to the time scale of the different dynamics of the population of the states composing the early dynamics of ISC.

**Figure 9:**
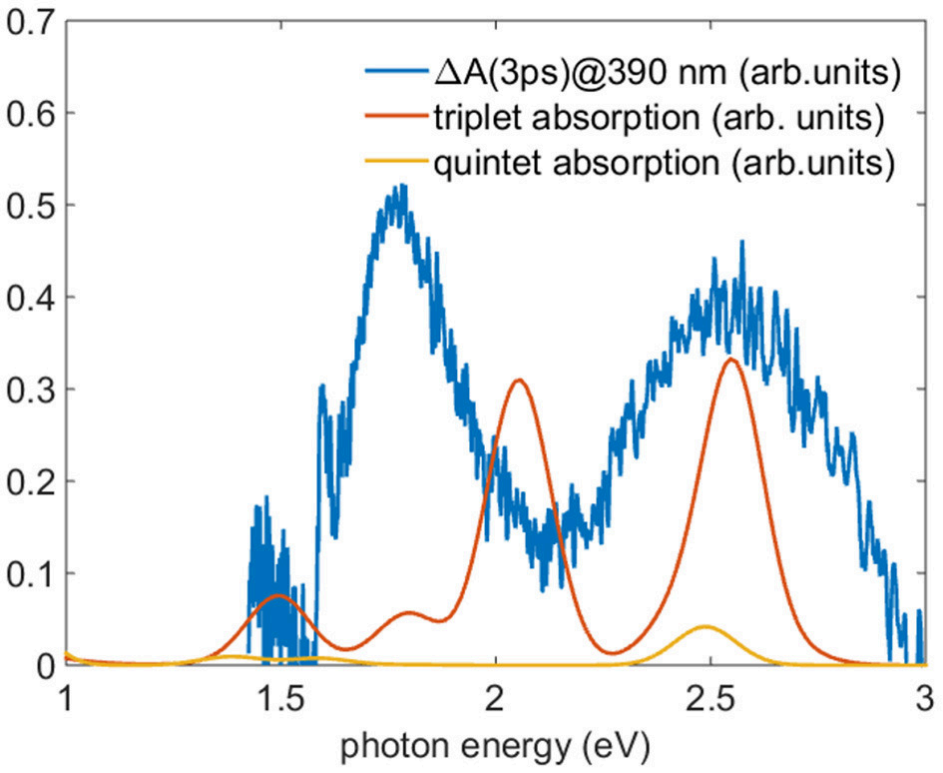
Comparison between the TA spectrum acquired 3 ps after the 390 nm excitation (blue line) and the TDDFT calculated absorption in acetonitrile of the lowest level of the triplet (red line) and quintet (yellow line). To reproduce the absorption the oscillator strength were convoluted with a gaussian function with standard deviation of 100 meV and the energy of the theoretical calculation shifted of 300 meV toward lower energy.

To test the hypothesis of the population of the lowest triplet and quintet levels, we compare the TA spectra after a time delay greater than 2 ps with the TDDFT calculations of the absorption spectra of the lowest energy levels of triplet and quintet states. Figure 9 reports the calculated spectra of triplet and quintet lowest states together with the experimental TA spectrum measured 3 ps after 390 nm excitation. The oscillator strengths are converted into absorption by convolution with a gaussian function with a standard deviation of 100 meV. In the comparison a rigid shift of 300 meV toward lower photon energies was applied. There is a clear accord in the branching ratio between the two most intense triplet transitions and the experimental curve. Although the absolute energy is slightly miscalibrated and the lack of vibrational calculations hinders the comparison, the energy difference of the calculated triplet transitions is 0.5 eV where the experimental energy difference between the experimental bands is about 0.7 eV, with a reasonable accord taking into account the accuracy of the TDDFT. This result sheds light on the process of the formation of the population of the states stemming from ISC suggesting the assignment of a predominant triplet character to the TA spectrum. According to these calculations, in the energy range investigated (1.5-3 eV) the ground state quintet oscillator strengths are one order of magnitude less favored than those of the triplets, and hence TA spectra are less sensitive to quintet transitions contributions. For this reason the presence of population in the quintet lowest level cannot be excluded.

VC occurs on the same time scale as the ISC and should be considered in the formation of the triplet (quintet) state. In the case of Co(AcAc)_3_, according to DFT calculations, the ground states of triplet and quintet states present an elongation of the Co-O bond length in the interval 0.1–0.2 Å associated with a lower symmetry with respect to the octahedral coordination. The elongation of the *R*_*Co*−*O*_ bond length is then related to the fastest processes. A similar elongation is found by means of X-ray TA for Fe-N bond of [Fe^II^(mbpy)_3_]^2+^ in acetonitrile for the photoinduced high spin (HS) ^5^T_2_ state (Liu et al., 2017).

**Figure 10:**
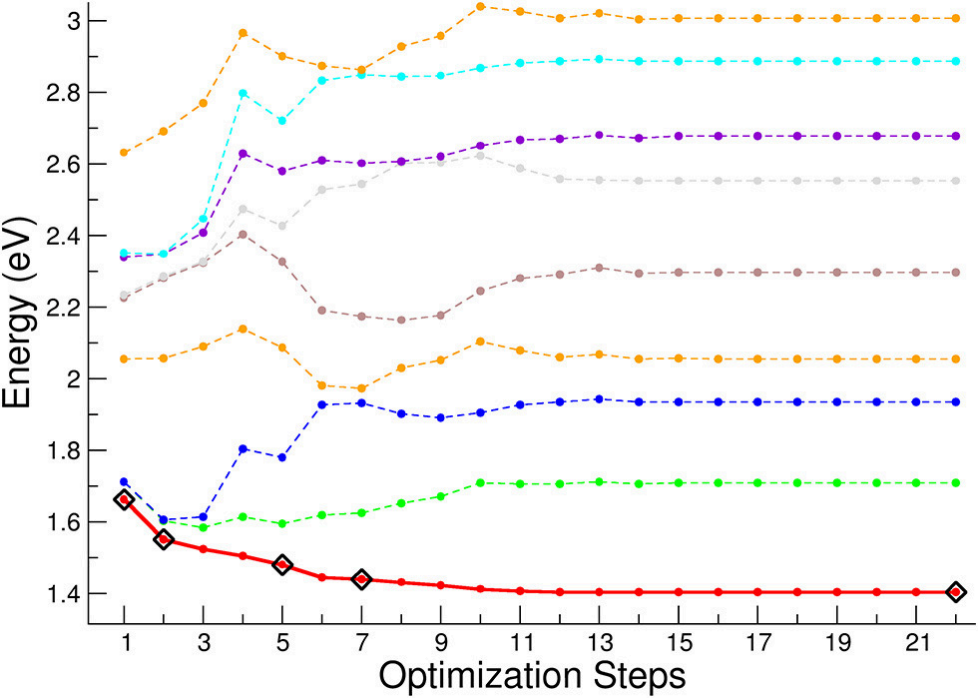
First singlet electronic excited state optimization. The energy is referred to the equilibrium geometry of the electronic ground state. The black rhombuses indicate the geometries where the spin-orbit coupling and ISC lifetimes have been calculated. In red thick line is reported the first singlet excited state, whereas with dashed lines are reported the lowest eight singlet electronic excited states.

In order to study the relaxation following the excitation processes, we have reported the geometrical optimization of the first singlet excited state in Figure 10. The higher energy curves (shown as colored lines) refer to higher singlet electronic states. The optimization steps corresponding to the sample geometry structures used to compute the spin-orbit coupling matrix and ISC lifetimes are shown as rhombuses. Here, the first point of the optimization steps represents the vertical excitation corresponding to the geometry of the equilibrium structure of the ground singlet state. It can be seen that the initial degeneracy, associated with the D_3_ symmetry, is removed during the relaxation path. After the 7th point of the optimization steps the energy remains almost constant, with a variation less than 30 meV with respect to the asymptotic energy corresponding to the equilibrium structure of the first excited singlet state. The higher energy singlet curves have a complex behavior along the relaxation path, and are characterized by several crossings, which open the way to a non-radiative decay by conical intersection from the singlet high energy states toward the lower excited singlet states.

**Figure 11:**
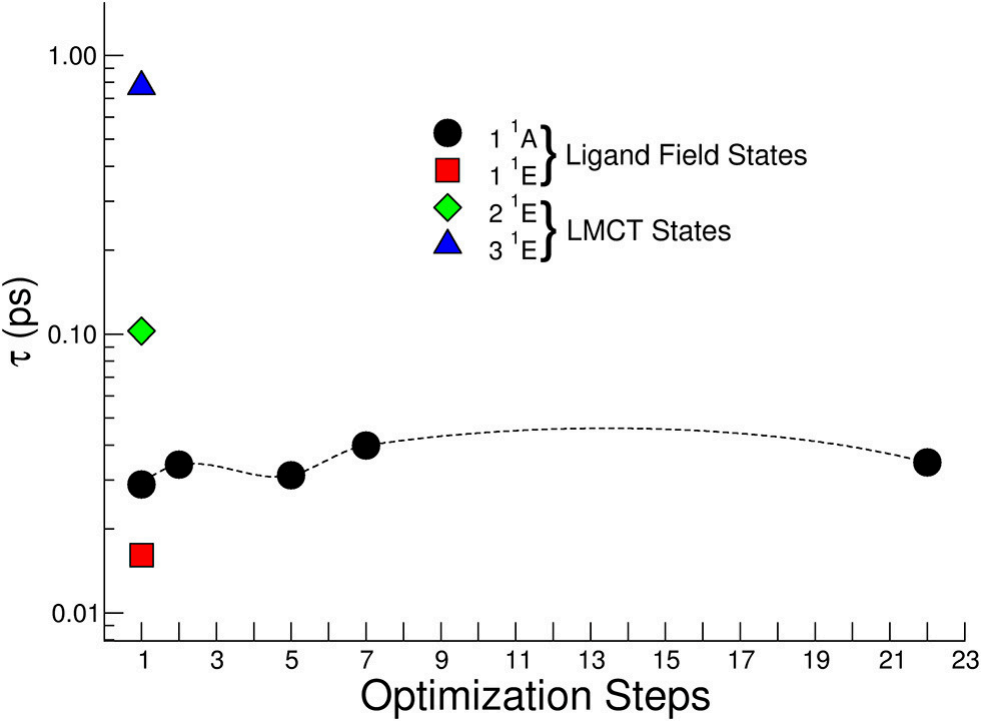
Non-radiative lifetimes for singlet-triplet intersystem crossing for several excited electronic states calculated at the geometry of the vertical transition (optimization step 1). For the 1 ^1^A state the dependence of ISC lifetimes on the optimization geometry steps is reported.

Figure 11 reports the calculated lifetime τ for the singlet – triplet ISC of the optically active first four singlet excited states calculated at the geometry of the vertical transition (step 1 in Figure 11). Only for the 1 ^1^A state the dependence of ISC lifetimes on the optimization steps is reported. The 1 ^1^A ISC lifetime weakly depends on the optimization step and beyond step 7 there is no substantial change in the geometry of the complex, and consequently the change in the spin-orbit coupling matrix is negligible. It is worth noticing that for the excited states corresponding to the LMCT the τ related to the vertical transition geometry is within the range 0.1 – 1.0 ps, while in the ligand field case is faster and presents values in the range 0.01-0.02 ps.

The ISC calculations performed offer a quantitative basis for the analysis of the experimental lifetimes.

The fast experimental lifetime τ_1_ = 0.08 ± 0.04 ps after excitation at 390 nm is in good agreement with that expressed by the 2 ^1^E state about 0.1 ps, suggesting a fast singlet-triplet ISC.

The 3 ^1^E state presents a τ about 1 ps at the geometry of the vertical transition and the measured lifetime τ_1_ = 0.07 ± 0.04 ps at 365 nm suggests an IC transition toward the first excited singlet state and then a fast ISC with lifetime about 20 fs from 1 ^1^A. However, we cannot exclude a fast geometry relaxation on this particular electronic state that lower the ISC lifetime to tens of fs. The dynamics expressed by the time constant τ_2_ contains the last part of the dynamics, the relaxation toward the lowest levels of the triplet state and, successively, to the ground states. Although, as explained above, the excited states populated with the pumps at 390 nm and 365 nm present a different interpretation in the dynamics, the information condensed in the time constants does not present a difference within experimental error.

**Figure 12:**
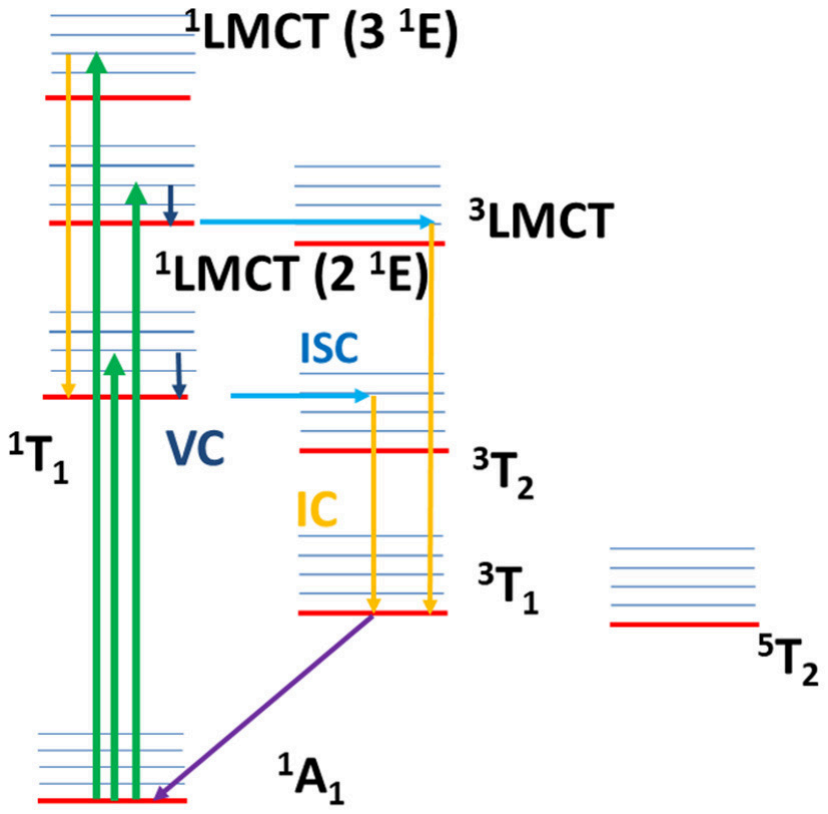
Jablonski diagram with the sketch of the excited state dynamics. The arrows explain qualitatively the dynamics: absorption transitions (green arrows), internal conversion (yellow arrows), intersystem crossing (light blue arrows), vibrational cooling (dark blue arrows), non-radiative decay toward the ground state (violet arrow). After ligand field excitation (^1^T_1_), VC and ISC occur with an overall lifetime 0.07 ± 0.04 ps and, successively, IC toward the lower triplet state and relaxation toward the ground state with a lifetime 1.8 ± 0.3 ps. The same dynamics is proposed for the ^1^LMCT (2 ^1^E) excitation, with VC and ISC (^1^LMCT → ^3^LMCT) overall lifetime 0.08 ± 0.04 ps, and IC and non-radiative relaxation overall lifetime 1.5 ± 0.3 ps. For ^1^LMCT (3 ^1^E) excitation the calculations assign an ISC lifetime about 1 ps, slower than the experimental rise time 0.07 ± 0.04 ps. A fast IC toward the first singlet excited state and a fast singlet-triplet ISC is suggested to be associated with the fast experimental lifetime. The decay toward the ground state is sketched by IC within the triplet states and non-radiative decay with lifetime 1.5 ± 0.3 ps.

The proposed dynamics is summarized in the Jablonski diagram reported in Figure 12, where the relaxation paths of ^1^LMCT (2 ^1^E) and ^1^LMCT (3 ^1^E) are shown.

To complete the study of the dynamics with excited states associated to the ligand-field excitation, the same analysis was performed for pump wavelengths of 580 and 650 nm. The spectral features of the absorption difference, plotted at different delay time (Figure 7 lower panels), have a comparable behavior with the 390 and 365 nm analysis; two broad bands are present and positive values in the investigated range. In particular, the TA spectra measured after 580 nm excitation exhibit the same qualitative evolution of the branching ratio of the LMCT excited spectra for a time delay greater than 2 ps. For the spectra obtained with a pump at 650 nm the branching ratio analysis is hampered by the presence of the residual pump signal. However, the band at lower wavelength appears to be extended toward the blue with respect to the LMCT case. Table 3 reports the time constants of the two processes as a function of selected probe energies for pump wavelengths of 580 and 650 nm, respectively. It is worth noticing that the fast rise measured in the previous case (τ_1_ = 0.07 ± 0.04 ps) is observed only in the band centered at about 700 nm, while it is within the temporal resolution of the experimental system in the band in the range 450–600 nm.

At excitation wavelength of 580 nm the slow lifetime constant associated to the averaged fit values is τ_2_ = 1.6 ± 0.2 ps for the low wavelength band (450–600 nm) and τ_2_ = 2.0 ± 0.2 ps for the high wavelength band (650–750 nm). For excitation at 650 nm the averaged lifetime constants are τ_2_ = 1.5 ± 0.2 ps and τ_2_ = 2.1 ± 0.2 ps for the low and high wavelength bands, respectively.

It is impossible to disentangle the decay time constant of the two relaxation channels and we assign an overall averaged value to the decay time constant in the ligand field excitation case τ_2_ = 1.8 ± 0.3.

The calculated singlet – triplet ISC τ for the ligand field states is in the range of 10 – 20 fs and confirms this phenomenological picture. In the low wavelength band the rise time is faster than the time resolution of the system and is compatible with an ISC of few tens of fs, while the high wavelength band probably contains information about VC relaxation.

The relaxation channels above discussed are reported in the Jablonski diagram in Figure 12 (^1^T_1_ excitation).

Comparing the results obtained in this study on Co(AcAc)_3_ with the Cr(AcAc)_3_ (Juban and McCusker, 2005) case, the dynamics of these molecules exhibits similar time constants and interpretations. Indeed, the order of magnitude of the time scale of the ISC presents a slight dependence on the different electronic and spin character of the transition (quadruplet to doublet in Cr(AcAc)_3_ and singlet to triplet in Co(AcAc)_3_).

## Conclusions

The FTAS study of Co(AcAc)_3_ displayed a very fast de-excitation dynamics characterized by a distribution of processes with lifetime constants on the scale 1-2 ps.

On the grounds of the TDDFT analysis we identified different processes associated with these dynamics. An elongation of Co-O bond length with reduction of octahedral symmetry subsequent to the LMCT excitation can be associated with the early stage of dynamics. The transient absorption spectra for delay times greater than 2 ps reveal the features of the absorption of the lowest triplet state. It is worth noticing that the findings of TDDFT theory can help to connect the phenomenological analysis to a microscopic model. A TDDFT calculation and a parametrized model was used to calculate singlet-triplet ISC lifetime at the vertical geometry, with values in the range 10-20 fs for LF states and 0.1-1 ps for LMCT states.

To summarize the dynamics, the formation of the population of the triplet involves different concomitant processes, such as vibrational relaxation, geometry distortion, intersystem crossing. The microscopic processes included in the fast time constant are the vibrational cooling and the intersystem crossing, while the slower lifetime decay involves the journey toward the lowest triplet state and the final relaxation to the ground states.

We believe that the characterization of the dynamics of metal complexes is desirable in view of possible spintronic applications that nano-fabrication could provide. The LMCT fast de-excitation with a short transient in the triplet state could be exploited in single molecule devices such as spin-valves.

## Author Contributions

MS and AP performed the theoretical calculations. The remaining authors performed the experiments and the data analysis. All the listed authors equally contributed to the scientific discussion and the writing of the manuscript.

### Conflict of Interest Statement

The authors declare that the research was conducted in the absence of any commercial or financial relationships that could be construed as a potential conflict of interest. The handling editor declared a past co-authorship with one of the authors DC.
